# Stramonium in Incipient Trismus

**Published:** 1840-09

**Authors:** 


### STRAMONIUM IN INCIPIENT TRISMUS.

Dr. Brice, whom we have just quoted, was called to a man who had received a wound of the scalp two days before, and found his jaws immovable, and other portons of his muscular system than the maxillary showed incipient tetanus. In the course of two hours, the doctor gave him two tea spoonfuls of the powdered seeds of stramonium; immediately afterwards the spasms ceased. D.

